# *ARID1A* knockdown enhances carcinogenesis features and aggressiveness of Caco-2 colon cancer cells: An *in vitro* cellular mechanism study

**DOI:** 10.7150/jca.65511

**Published:** 2022-01-01

**Authors:** Paleerath Peerapen, Kanyarat Sueksakit, Wanida Boonmark, Sunisa Yoodee, Visith Thongboonkerd

**Affiliations:** Medical Proteomics Unit, Office for Research and Development, Faculty of Medicine Siriraj Hospital, Mahidol University, Bangkok 10700, Thailand.

**Keywords:** Chemoresistance, Colorectal cancer, Invasion, Proliferation, Spheroid, VEGF

## Abstract

Loss of *ARID1A*, a tumor suppressor gene, is associated with the higher grade of colorectal cancer (CRC). However, molecular and cellular mechanisms underlying the progression and aggressiveness of CRC induced by the loss of *ARID1A* remain poorly understood. Herein, we evaluated cellular mechanisms underlying the effects of *ARID1A* knockdown on the carcinogenesis features and aggressiveness of CRC cells. A human CRC cell line (Caco-2) was transfected with small interfering RNA (siRNA) specific to *ARID1A* (siARID1A) or scrambled (non-specific) siRNA (siControl). Cell death, proliferation, senescence, chemoresistance and invasion were then evaluated. In addition, formation of polyploid giant cancer cells (PGCCs), self-aggregation (multicellular spheroid) and secretion of an angiogenic factor, vascular endothelial growth factor (VEGF), were examined. The results showed that *ARID1A* knockdown led to significant decreases in cell death and senescence. On the other hand, *ARID1A* knockdown enhanced cell proliferation, chemoresistance and invasion. The siARID1A-transfected cells also had greater number of PGCCs and larger spheroid size and secreted greater level of VEGF compared with the siControl-transfected cells. These data, at least in part, explain the cellular mechanisms of *ARID1A* deficiency in carcinogenesis and aggressiveness features of CRC.

## Introduction

*ARID1A* encoding adenine-thymine (AT)-rich interactive domain-containing protein 1A (ARID1A), also known as BAF250a, SMARCF1 and p27, is recognized as a tumor suppressor gene 1-3. ARID1A protein is a subunit of SWI/SNF (SWItch/Sucrose Non-Fermentable) complex. Normally, this complex plays roles in regulation of gene expression via ATP hydrolysis-dependent nucleosome remodeling to make DNA accessible during transcription, replication and DNA repair 1-3. Mutations and loss of genes encoding proteins in this complex have been found in several cancers 4-6. In particular, *ARID1A* is frequently mutated and/or lost in various cancers, including ovarian, breast, and hepatic cancers 7-12. Additionally, the loss of *ARID1A* tumor suppressor gene is associated with the higher cancer mortality rate 13, 14.

Colorectal cancer (CRC) is considered as a lethal disease. The global incidence of this cancer type (10% of diagnosed cases) and its mortality rate (9.4% of dead cases) are ranked numbers three and two, respectively, among all cancers 15. Although the survival rate of this cancer at an early stage is comparable to other cancers, its mortality rate is very high, especially in patients with higher stages 15. Therefore, better understanding of the disease pathogenesis and pathophysiologic processes that regulate CRC progression may be helpful for lowering the incidence and mortality rate of CRC. Interestingly, a recent meta-analysis has found mutations and loss of *ARID1A* in 13% and 11.7%, respectively, of the CRC patients 16. In concordance, a more recent study has shown that 12/18 (approximately 67%) of CRC tissues had no or low *ARID1A* expression 17. Additionally, several previous studies using human specimens or animal models have reported an association between *ARID1A* down-regulation and CRC tumorigenicity 18-22. Moreover, the loss of *ARID1A* is associated with the poorly differentiated grade of CRC cells 16. Nonetheless, cellular mechanisms underlying the progression and aggressiveness of CRC induced by the loss of *ARID1A* remain poorly understood.

This study therefore evaluated cellular mechanisms underlying the effects of *ARID1A* knockdown on the carcinogenesis features and aggressiveness of CRC. Caco-2 cells were transfected with small interfering RNA (siRNA) specific to *ARID1A* (siARID1A) or non-specific siRNA (siControl). Thereafter, several various assays were performed to examine cell death, proliferation, senescence, chemoresistance, invasion, polyploid giant cancer cells (PGCCs) formation, self-aggregation, and secretion of an angiogenic factor VEGF (vascular endothelial growth factor), in the si-ARID1A-transfected cells compared with those transfected with siControl.

## Materials & Methods

### Cell culture

Caco-2, a human colon adenocarcinoma cell line (ATCC; Manassas, VA), was grown in the growth medium containing Dulbecco's Modified Eagle's medium (DMEM) (Gibco; Grand Island, NY) supplemented with 10% fetal bovine serum (FBS) (Gibco), 1% non-essential amino acid (Sigma-Aldrich; St. Louis, MO), 60 U/ml Penicillin G (Sigma-Aldrich) and 60 µg/ml Streptomycin (Sigma-Aldrich). The cells were maintained in a humidified incubator with 5% CO_2_ at 37°C.

### *ARID1A* knockdown by siRNA

siRNA transfection was performed using a protocol reported previously 23, 24. Briefly, the cells were seeded in each well of the 6-well plate (Corning Inc.; Corning, NY) and grown in antibiotics-free growth medium containing 10% FBS overnight. siRNA specific to *ARID1A* (siARID1A) (Santa Cruz Biotechnology; Santa Cruz, CA) or control siRNA consisting of a scrambled sequence (siControl) (Santa Cruz Biotechnology) was premixed with siRNA Transfection Reagent (Santa Cruz Biotechnology) in Opti-MEM (Gibco) and incubated at 25 °C for 45 min. An equal dose (40 pmol) of siARID1A or siControl was then added and incubated with the cells at 37 °C in a humidified incubator with 5% CO_2_ for 6 h. Thereafter, the cells were further incubated in complete growth medium for 48 h prior to all subsequent functional investigations as follows.

### Semi-quantitative reverse transcription polymerase chain reaction (RT-PCR)

Total RNA was extracted from siControl-transfected and siARID1A-transfected cells using Trizol reagent (Invitrogen; Carlsbad, CA) and Direct-zol RNA MiniPrep (Zymo Research; Irvine, CA). An equal amount of total RNA was used for preparation of cDNA with Super Script III reverse transcriptase (Invitrogen). Semi-quantitative RT-PCR was performed using Taq DNA polymerase (New England BioLabs; Beverly, MA) to assess mRNA expression level of *ARID1A*, whereas the mRNA level of *GAPDH* served as the loading control. The amplification was carried out using the following primers: *ARID1A* forward: 5'-CCCCTCAATGACCTCCAGTA-3', reverse: 5'-CTGGAAATCCCTGATGTGCT-3'; *GAPDH* forward: 5'-CATCACTGCCACCCAGAAGA-3', reverse: 5'-GTGTAGCCCAGGATGCCTTT-3'.

The PCR reaction was started with initial DNA denaturation step (at 95 °C for 3 min) followed by 30 cycles of denaturation at 95 °C for 30 sec, annealing at 55 °C for 30 sec and extension at 72 °C for another 30 sec. The PCR products were then resolved by 1.5% agarose gel electrophoresis and stained with ethidium bromide. The DNA bands were visualized using ChemiDoc MP Imaging System (Bio-Rad; Berkeley, CA) and quantitated by using ImageQuant TL software (GE Healthcare; Uppsala, Sweden).

### Western blot analysis

Proteins were extracted from individual samples using Laemmli's buffer and their concentrations were measured by Bradford's method using Bio-Rad Protein Assay (Bio-Rad). Proteins with an equal amount (50 µg/sample/lane) were resolved by 12% SDS-PAGE and transferred onto a nitrocellulose membrane. After blocking non-specific bindings with 5% skim-milk/PBS for 1 h, the membrane was incubated at 4 °C overnight with mouse monoclonal anti-ARID1A (Santa Cruz Biotechnology) or mouse monoclonal anti-GAPDH (Santa Cruz Biotechnology) (all were diluted 1:1,000 in 1% skim milk/PBS). After probing with corresponding secondary antibody conjugated with horseradish peroxidase (HRP) (Dako; Glostrup, Denmark) (diluted 1:2,000 in 1% skim milk/PBS) at 25 °C for 1 h, the immunoreactive protein bands were visualized by SuperSignal West Pico chemiluminescence substrate (Pierce Biotechnology, Inc.; Rockford, IL) and autoradiography. Band intensity data was obtained using ImageQuant TL software (GE Healthcare).

### Immunofluorescence staining

The cells were grown on a coverslip and treated as described above. After rinsing with PBS, the cells were fixed with 4% (v/v) paraformaldehyde/PBS at 25 °C for 15 min and then permeabilized with 0.1% Triton X-100/PBS at 25°C for 15 min. After washing, non-specific bindings were blocked with 1% BSA in PBS at 25 °C for 30 min and the cells were incubated at 4 °C overnight with mouse monoclonal anti-ARID1A (Santa Cruz Biotechnology) (1:50 in 1% BSA/PBS). After another washing step, the cells were incubated with corresponding secondary antibody conjugated with Alexa Fluor 488 (Invitrogen) (1:2,000 in 1% BSA/PBS) at 25 °C for 1 h. Nuclei were counterstained by Hoechst dye (Sigma-Aldrich) (1:2,000 in 1% BSA/PBS). Finally, the cells were extensively washed with PBS and mounted onto a glass slide using 50% glycerol in PBS. Cellular imaging was done by using Nikon Eclipse 80i fluorescence microscope (Nikon; Tokyo, Japan) and expression level of ARID1A was analyzed by measuring mean fluorescence intensity from at least 100 cells in ≥10 random high-power fields (HPFs) of each sample using NIS-Elements D V.4.11 (Nikon).

### Flow cytometric analysis of cell death using annexin V-FITC/propidium iodide co-staining

The cells were detached from cell monolayers in the culture well using 0.1% trypsin in 2.5 mM EDTA/PBS and washed twice with ice-cold PBS. The cell pellets were then resuspended with annexin V buffer (10 mM HEPES, 140 mM NaCl and 2.5 mM CaCl_2_.2H_2_O; pH 7.4) (BD Biosciences; San Jose, CA) and further incubated with FITC-labelled annexin V (BD Biosciences) on ice in the dark for 15 min. Propidium iodide (BD Biosciences) at the final concentration of 0.2 µg/ml was added to the cell suspension prior to analysis by using a flow cytometer (BD Accuri C6) (BD Biosciences) 25, 26.

### Total cell count

All the cells in each well were detached by incubation with 0.1% trypsin in 2.5 mM EDTA/PBS and were harvested. The cells pellet was collected by centrifugation at 1,000 *g* for 5 min. After resuspended in PBS, the cells were stained with 0.4% trypan blue solution (Gibco). Thereafter, total cell number was counted using a hemacytometer under a phase contrast light microscope (CKX41; Olympus, Tokyo, Japan).

### Cellular senescence assay

Cellular senescence was evaluated by cytochemical detection of the senescence-associated beta-galactosidase (SA-β-gal) activity. Briefly, the cells were rinsed twice with PBS before incubation with a fixative solution (2% formaldehyde and 0.2% glutaraldehyde in PBS) at 25 °C for 5 min. After removing the fixative solution and further rinsing with PBS, the cells were then incubated with the staining solution (40 mM citric acid/Na phosphate buffer, 5 mM K_4_[Fe(CN)_6_]·3H_2_O, 5 mM K_3_[Fe(CN)_6_], 150 mM NaCl, 2 mM MgCl_2_, and 1 mg/ml X-gal) at 37 °C for 16 h. The staining solution was then discarded and the cells were washed twice with PBS followed by another wash with methanol. After air dry, the cells were imaged under an inverted phase-contrast microscope (Eclipse Ti-S) (Nikon).

### Chemoresistance assay

Docetaxel (Hospira; Melbourne, Australia) was used as a chemotherapeutic drug to kill the cancer cells. The cells were treated with 10 mM docetaxel and maintained at 37 °C in a humidified incubator with 5% CO_2_ for 24 h. Thereafter, the cells were subjected to cell death assay by flow cytometry as described above.

### Cell invasion assay

The cells were cultured in serum-free growth medium for 24 h. In parallel, Matrigel (BD Biosciences) was pre-coated onto polycarbonate membrane insert (5-µm pore size) of Transwell cultured plate (0.33-cm^2^ culture area/well) (Corning Costar; Cambridge, MA) and further incubated at 37 °C in a humidified incubator with 5% CO_2_ for 24 h. Thereafter, the cells were detached from cell monolayers using 0.1% trypsin in 2.5 mM EDTA/PBS and resuspended in serum-free growth medium. The cell suspension (2×10^5^ cells in 200 μl medium) was added into upper chamber of each well, whereas the lower chamber of each well contained 500 μl complete growth medium (containing 10% FBS, as the source for chemoattractants). The Transwell plates were incubated at 37 °C in a humidified incubator with 5% CO_2_ for 24 h. Thereafter, the non-invading cells remained on the upper surface of the membrane inserts were swapped out and the membrane inserts were washed with PBS. The invading cells appeared on the lower surface of the membrane inserts were fixed with 3.7% (v/v) formaldehyde in PBS for 15 min and stained with Hoechst dye (1:2,000 in PBS) at 25 °C for 10 min. The stained cells were then imaged using the Nikon Eclipse 80i fluorescence microscope. Number of the invaded cells was counted from ≥10 random low-power fields (LPFs) for each group.

### Self-aggregation (hanging drop) assay

The cells were detached from cell monolayers in the culture well using 0.1% trypsin in 2.5 mM EDTA/PBS and resuspended in complete growth medium at 2.5×10^5^ cells/ml. The cell suspension was dropped (20 µl/drop) onto the inner side of upper lid of 100-mm tissue culture dish (10 drops/dish). The upper lid was then inverted to cover the dish containing 5 ml of the growth medium at the bottom for humidification. The dishes were incubated at 37 °C in a humidified incubator with 5% CO_2_ for 24 h. A total of 30 drops for each group were harvested, disbursed by pipetting up and down for several times, and examined for self-aggregated multicellular spheroid formation under an inverted phase-contrast microscope (Eclipse Ti-S) (Nikon). Size of the multicellular spheroid was measured from at least 100 aggregates using NIS-Elements D V.4.11 program (Nikon).

### ELISA

ELISA was performed to measure level of VEGF. The cells were cultured in serum-free growth medium for 24 h. The culture supernatant was then collected, lyophilized and resuspended in 500 μl deionized water. An equal amount of 2.5 μg total protein of each sample was mixed with a coating buffer containing 15 mM Na_2_CO_3_ and 30 mM NaHCO_3_ (pH 9.4) and then coated in each well of the 96-well ELISA plate (Nunc; Roskilde, Denmark). After an overnight incubation at 4 °C, the sample wells were washed five times with a washing buffer (0.05% Tween-20 in PBS). Non-specific bindings were blocked with 1% BSA/PBS at 25 °C for 2 h. After other five washes, mouse monoclonal anti-VEGF antibody (Santa Cruz Biotechnology) (1:1,000 in 0.1% BSA/PBS) was incubated with the samples at 25 °C for 2 h. After other five washes, the samples were then probed with rabbit anti-mouse IgG conjugated with horseradish peroxidase (1:2,000 in 0.1% BSA/PBS) at 25 °C for 1 h in the dark. After the final five washes, the reaction color was developed by incubation with 100 μl chromogenic substrate (1.5 mM ortho-phenylenediamine dihydrochloride (Sigma-Aldrich) in 35 mM citric acid (Bio-Basic; Markham, Canada) and 0.012% H_2_O_2_ (Fisher Scientific; Loughborough, UK) (pH 5.5) in the dark for 15 min. Finally, the reaction was stopped by adding 50 μl of 2 M H_2_SO_4_. Absorbance (optical density) of each sample was then measured at λ492 nm using an ELISA microplate reader (EZRead 400, Biochrom Ltd.; Cambridge, UK).

### Statistical analysis

All quantitative data were derived from at least three independent experiments and are reported as mean ± SEM. Comparisons between two groups were done by unpaired Student's t-test. P values < 0.05 were considered statistically significant.

## Results

### Effective down-regulation of *ARID1A* expression by siRNA knockdown

**Figure 1:**
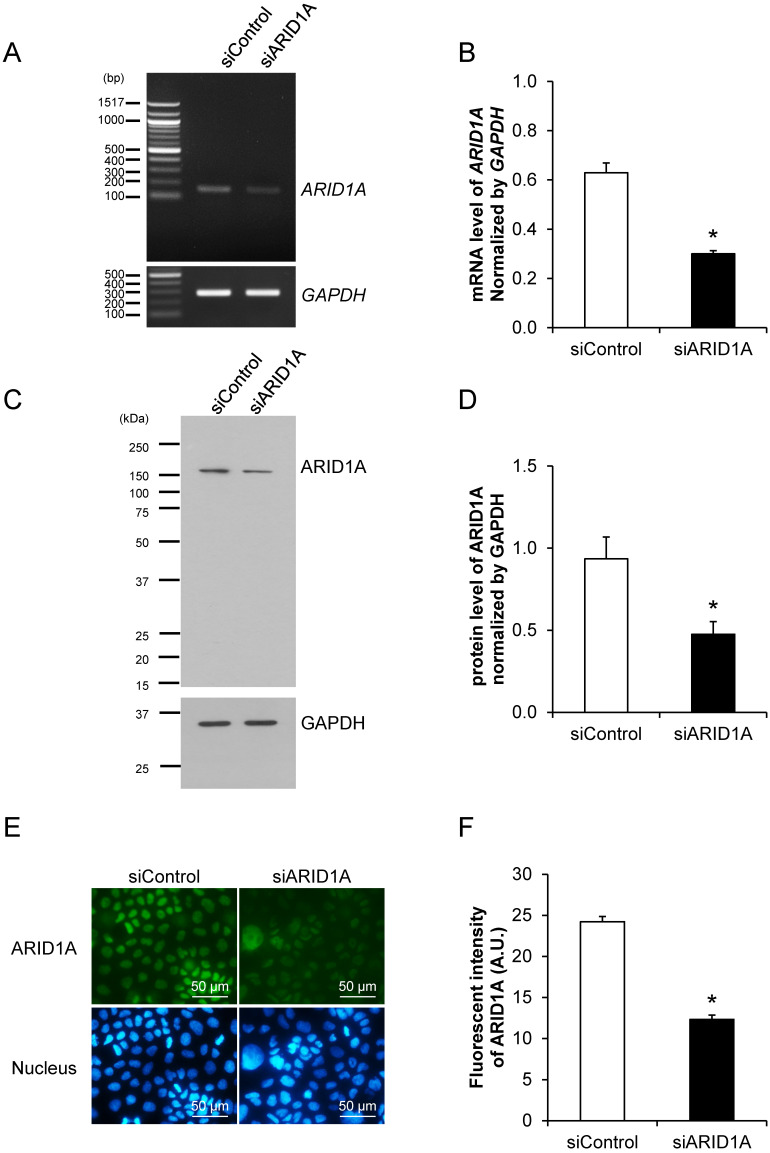
** Effective down-regulation of *ARID1A* expression by siRNA knockdown. (A-B)** Semi-quantitative RT-PCR to measure mRNA level of *ARID1A* normalized with that of *GAPDH*. **(C-D)** Western blot analysis of ARID1A protein. **(E-F)** Immunofluorescence staining of ARID1A protein. Intensities of the transcript and protein bands were quantitated by using ImageQuant TL software (GE Healthcare). Mean fluorescence intensity was measured from at least 100 cells in ≥10 random high-power fields (HPFs) of each sample using NIS-Elements D V.4.11 (Nikon). Each bar represents mean ± SEM of the data derived from three independent experiments. *p < 0.05 vs. siControl-transfected cells; A.U. = arbitrary fluorescence unit.

Prior to examining the effects of *ARID1A* knockdown on carcinogenesis features and aggressiveness of cancer cells, the efficacy of siRNA specific to ARID1A (siARID1A) was first examined. Semi-quantitative RT-PCR confirmed the decrease of *ARID1A* transcript by approximately 50% in the siARID1A-transfected cells compared with the siControl-transfected cells (**Figures 1A and 1B**). In concordance, Western blot analysis and immunofluorescence study both confirmed the decreased level of ARID1A protein by approximately a half (**Figures 1C-1F**) in the siARID1A-transfected cells. These data indicate the effective down-regulation of *ARID1A* by siRNA knockdown in Caco-2 cells.

### Effects of *ARID1A* knockdown on cell death and proliferation

**Figure 2:**
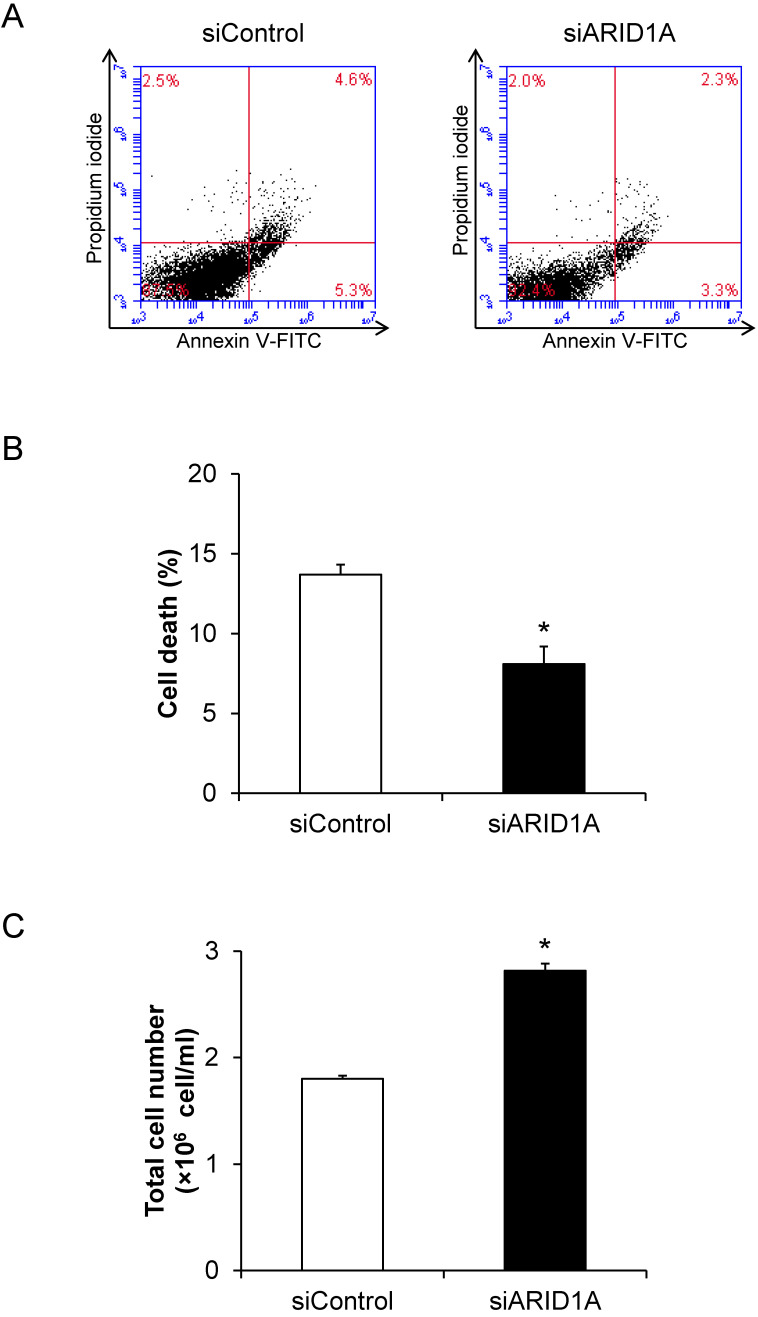
** Effects of *ARID1A* knockdown on cell death and proliferation. (A)** Scatter plot of the cells stained by annexin V-FITC and/or propidium iodide detected by flow cytometry. **(B)** Percentage of cell death. **(C)** Total cell number using hemacytometer. Each bar represents mean ± SEM of the data derived from three independent experiments. *p < 0.05 vs. siControl-transfected cells.

To determine the role for *ARID1A* down-regulation in cancer cell survival, flow cytometric analysis of cell death using annexin V-FITC/propidium iodide was performed. The scatter plot showed the results of annexin V-FITC and propidium iodide signals (**Figure 2A**). Quantitative analysis revealed the significantly lower cell death of the siARID1A-transfected cells compared with the siControl-transfected cells (**Figure 2B**). In concordance, the total cell number, which reflected cell proliferation, was significantly greater in the siARID1A-transfected cells (**Figure 2C**). These results indicated that the down-regulation of *ARID1A* inhibited cell death but, on the other hand, promoted cell proliferation.

### Effects of *ARID1A* knockdown on cellular senescence

**Figure 3:**
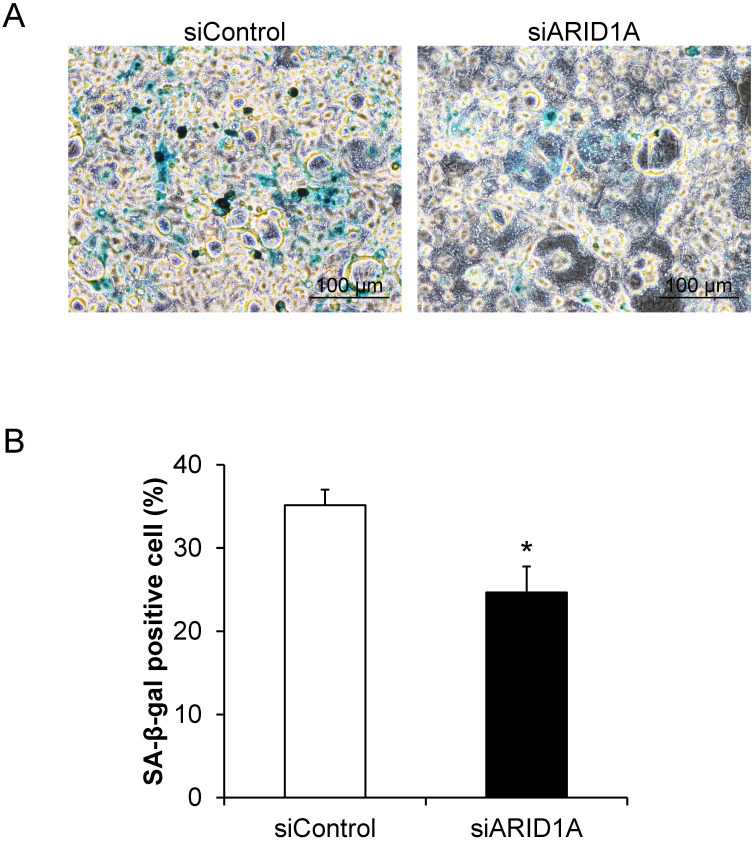
** Effects of *ARID1A* knockdown on cellular senescence. (A)** SA-β-gal-positive cells are shown in blue. **(B)** Percentage of the SA-β-gal positive cells was analyzed from at least 100 cells in ≥10 random high-power fields (HPFs) of each sample using NIS-Elements D V.4.11 (Nikon). Each bar represents mean ± SEM of the data derived from three independent experiments. *p < 0.05 vs. siControl-transfected cells.

Cellular senescence, which was determined by the senescence-associated beta-galactosidase (SA-β-gal) activity, was evaluated. The assay demonstrated the SA-β-gal-positive cells, which underwent senescence process, in blue (**Figure 3A**). Quantitative analysis revealed that percentage of the SA-β-gal-positive cells in the siARID1A-transfected cells was significantly lower than that of the siControl-transfected cells (**Figure 3B**). This data indicated that the down-regulation of *ARID1A* suppressed cellular senescence.

### Effects of *ARID1A* knockdown on chemoresistance

**Figure 4:**
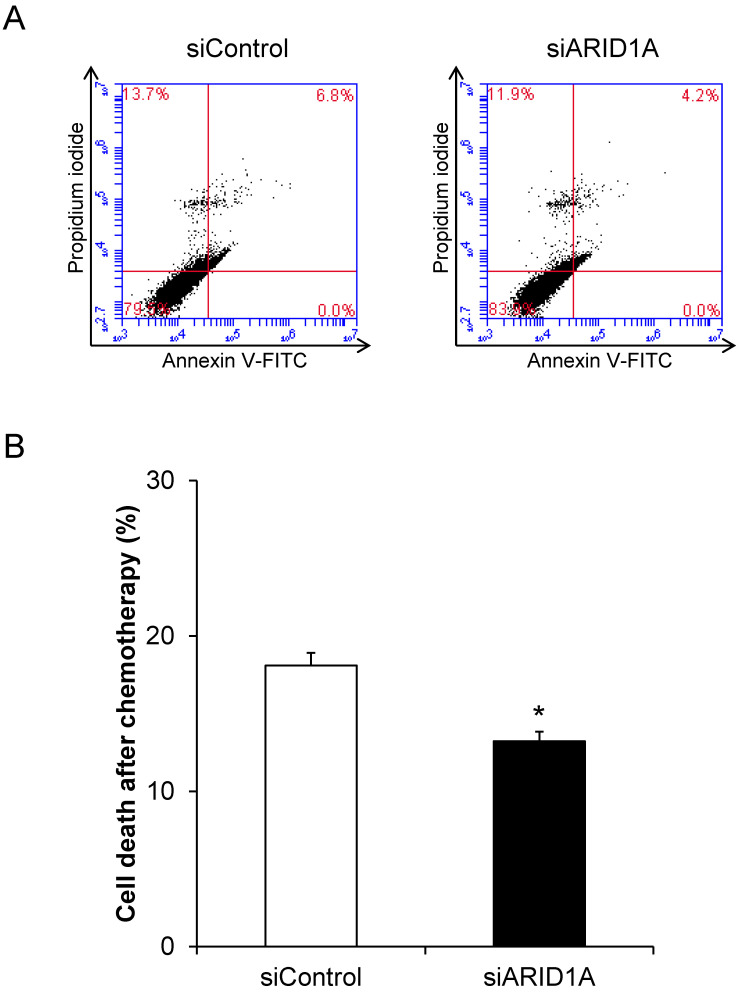
** Effects of *ARID1A* knockdown on chemoresistance. (A)** Scatter plot of the cells stained by annexin V-FITC and/or propidium iodide detected by flow cytometry after treatment with 10 mM docetaxel for 24 h. **(B)** Percentage of cell death after treatment with 10 mM docetaxel for 24 h. Each bar represents mean ± SEM of the data derived from three independent experiments. *p < 0.05 vs. siControl-transfected cells.

To evaluate the chemoresistance, which is one of the features of cancer cells, docetaxel was used. Flow cytometric analysis using annexin V-FITC/propidium iodide revealed significant decrease of cell death in the siARID1A-transfected cells after treatment with docetaxel compared with the treated siControl-transfected cells (**Figure 4**). This data indicated that the down-regulation of *ARID1A* enhanced chemoresistance of the cancer cells.

### Effects of *ARID1A* knockdown on cell invasion

**Figure 5:**
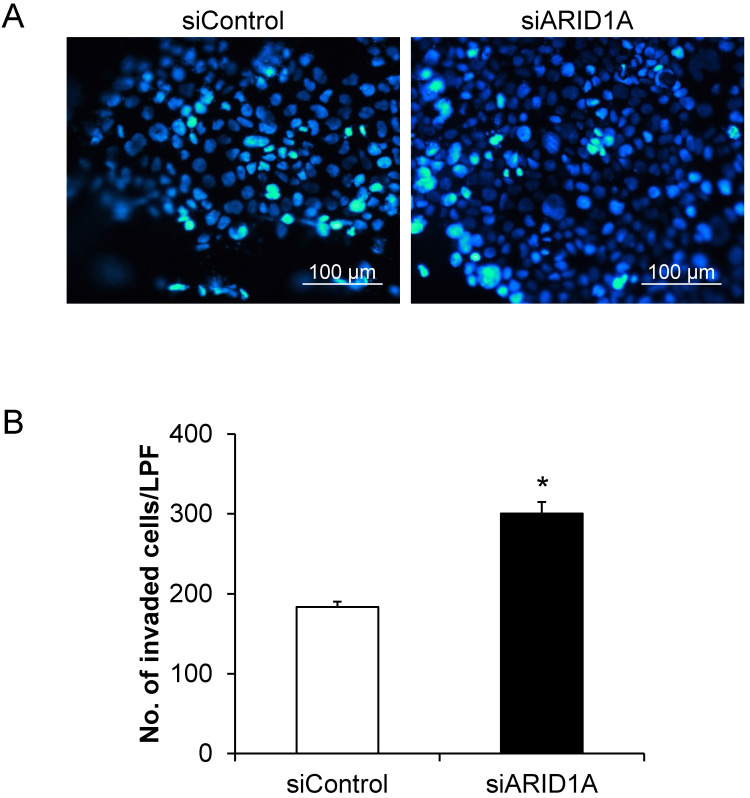
** Effects of *ARID1A* knockdown on cell invasion. (A)** The invaded cells attached at the bottom side of the Transwell membrane were stained with Hoechst dye (shown in blue). **(B)** Number of the invaded cells was counted from ≥10 random low-power fields (LPFs) for each group. Each bar represents mean ± SEM of the data derived from three independent experiments. *p < 0.05 vs. siControl-transfected cells.

Cell invasion capability was evaluated using the Transwell-based invasion assay. Number of the cells that passed (invaded) through the Transwell membrane sieves was counted. **Figure 5A** shows the Hoechst-stained nuclei of the invaded cells. The quantitative data showed that the siARID1A-transfected cells had significantly greater number of the invaded cells compared with the siControl-transfected cells (**Figure 5B**). This data indicated that the down-regulation of *ARID1A* promoted invasion capability of the cancer cells.

### Effects of *ARID1A* knockdown on polyploid giant cancer cells (PGCCs) formation

**Figure 6:**
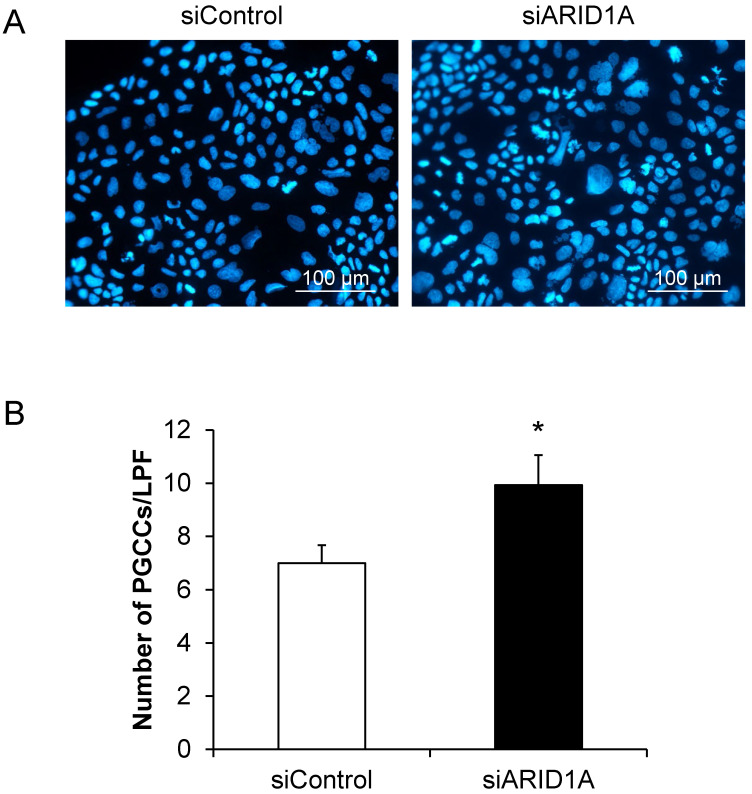
** Effects of *ARID1A* knockdown on polyploid giant cancer cells (PGCCs) formation. (A)** The fluorescence image of PGCCs stained with Hoechst dye (shown in blue). **(B)** Number of the PGCCs was counted from ≥10 random low-power fields (LPFs) for each group. Each bar represents mean ± SEM of the data derived from three independent experiments. *p < 0.05 vs. siControl-transfected cells.

PGCCs formation is one of the indicators for aggressiveness and poor prognosis of cancers. The PGCCs number was thus counted and compered between the two groups. **Figure 6A** shows that some of the cancer cells had giant nuclei and were thus counted as the PGCCs. Quantitative analysis revealed significant greater number of the PGCCs in the siARID1A-transfected cells compared with those transfected with siControl (**Figure 6B**). This data suggested that the down-regulation of *ARID1A* increased the aggressiveness of the cancer cells.

### Effects of *ARID1A* knockdown on self-aggregation of the cells or formation of multicellular spheroid

**Figure 7:**
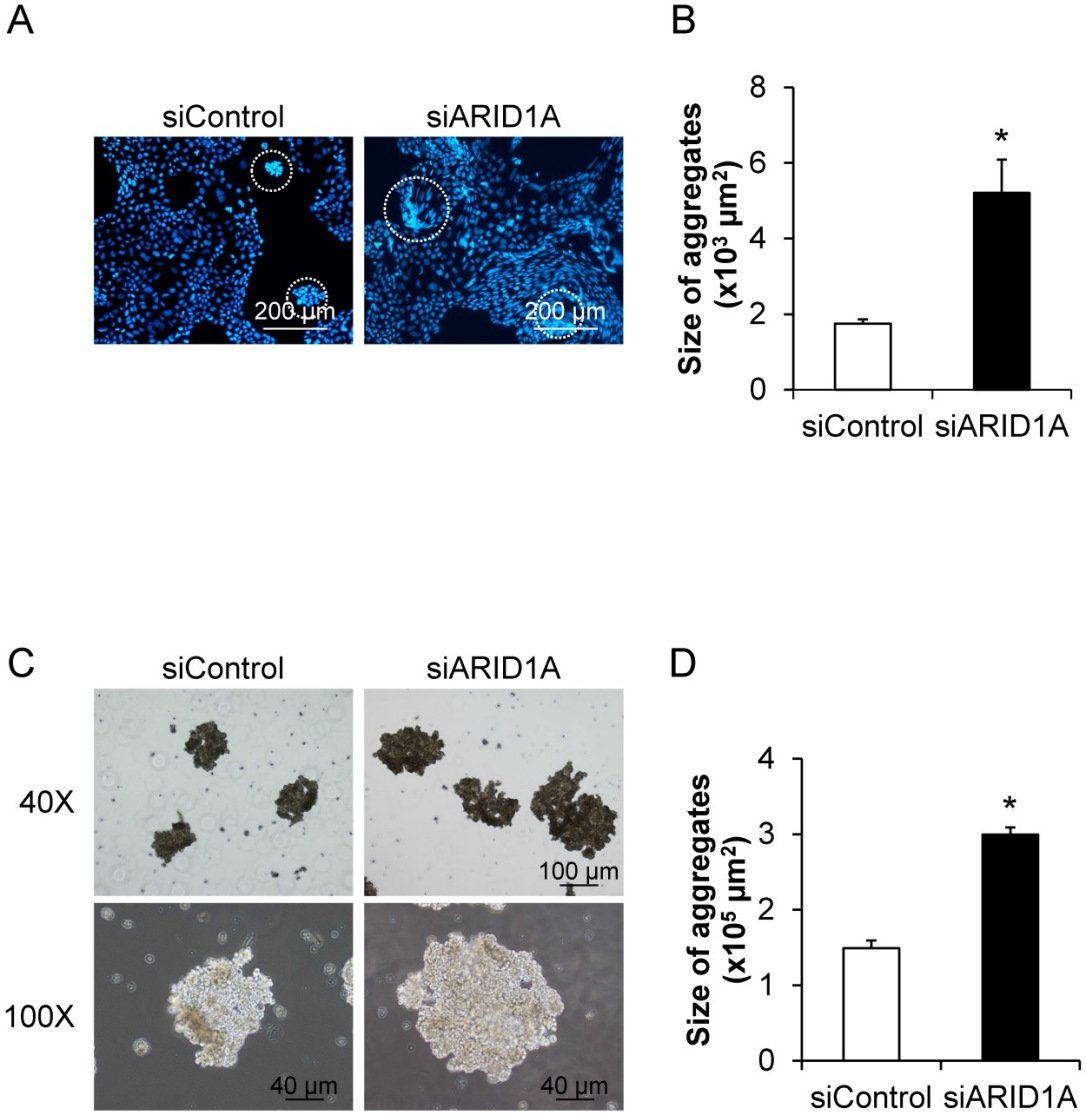
** Effects of *ARID1A* knockdown on self-aggregation of the cells or formation of multicellular spheroid. (A)** The fluorescence image of self-aggregates of the cancer cells stained with Hoechst dye (shown in blue). **(C)** Self-aggregates of the cancer cells were also examined using the hang-drop assay. **(B and D)** Size of the multicellular spheroid was measured from at least 100 aggregates using NIS-Elements D V.4.11 (Nikon). Each bar represents mean ± SEM of the data derived from three independent experiments. *p < 0.05 vs. siControl-transfected cells.

To determine the effects of *ARID1A* knockdown on cellular self-aggregation or formation of multicellular spheroid, we applied two methods. The first one was based on fluorescence staining, in which the nuclei were stained with Hoechst dye and observed under a fluorescence microscope (**Figure 7A**). The second method was the non-fluorescence hanging drop assay (**Figure 7C**). Both methods provided the consistent data demonstrating the significant larger size of the cancer cell spheroid in the siARID1A-transfected cells compared with the siControl-transfected cells (**Figures 7B and 7D**). These data indicated that the down-regulation of *ARID1A* promoted cancer cell self-aggregation.

### Effects of *ARID1A* knockdown on VEGF secretion

**Figure 8:**
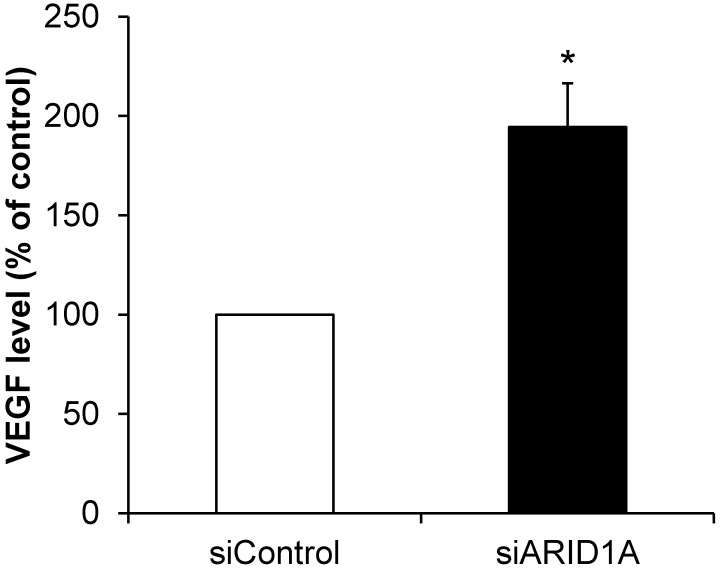
** Effects of *ARID1A* knockdown on VEGF secretion.** Level of VEGF secreted into culture supernatant was measured by ELISA. Each bar represents mean ± SEM of the data derived from three independent experiments. *p < 0.05 vs. siControl-transfected cells.

Angiogenesis is one of the features of carcinogenesis and cancer aggressiveness. To investigate the effects of *ARID1A* knockdown on the ability of cancer cells to induce angiogenesis, level of secretory VEGF was measured using an ELISA assay. The data showed significantly greater level of VEGF secreted from the siARID1A-transfected cells as compared with that from the siControl-transfected cells (**Figure 8**). This data implicated that the down-regulation of *ARID1A* induced or enhanced angiogenesis.

## Discussion

The cancer cells are usually graded based on degree of their differentiation 27-29. The cells that are well organized and apparently similar to the normal cells are defined as low-grade (well-differentiated) cells, whereas those with disorganization are defined as high-grade (poor-differentiated or undifferentiated) cells. In addition, the poor-differentiated grade is associated with the aggressive behaviors of cancer cells 27-29. Aggressiveness of the cancer cells is characterized by chemoresistance toward various chemotherapy agents, increased cell proliferation, migration, invasion, etc. 30, 31. The aggressive phenotypes of CRC cells are also associated with enhancement of the hallmark of carcinogenesis, including resisting cell apoptosis, sustaining proliferation signal, enabling replicative immortality, evading growth suppressor, activating invasion, and inducing angiogenesis 32.

As aforementioned, there are several previous studies demonstrating the association between *ARID1A* down-regulation and CRC in human tissues or animal models 16-22. Herein, we thus focused our attention on the cellular mechanism study, whereas a validation to demonstrate the down-regulation of *ARID1A* expression in CRC tissues was not done. Caco-2 cell line was originated from a 72-year-old Caucasian male with primary colon adenocarcinoma 33. In nude mice, the tumorigenicity of this cell line is generally classified as the moderately well-differentiated phenotype (consistent with grade II primary CRC) 33-36. Several previous studies had employed this cell line to investigate the effects of various inducers on the progression of CRC or its tumorigenicity 37-40. Additionally, Caco-2 is a CIMP (CpG island methylator phenotype) positive cancer cell 41, 42. Interestingly, the down-regulation of *ARID1A* in several cancer types has been shown to be associated with the promoter hypermethylation in CpG island of the gene 17, 43. We thus used only this cell model to address cellular mechanisms underlying the significant roles of *ARID1A* down-regulation in the carcinogenesis and aggressiveness features of CRC. It should be noted that these cellular mechanisms may not entirely explain all types of CRC.

We first confirmed the association between the down-regulation of *ARID1A* and resistance to apoptosis (or the programmed cell death), which is required for normal cell turnover and tissue homeostasis. Resistance to the cell death is one of the important characteristic features of the cancer cells. Additionally, the association between high-grade staging and cell death resistance has been reported in several cancers 44-47. Interestingly, inactivation of SWI/SNF complex by *ARID1A* mutation has been reported to be associated with the decreased cell death in ovarian cancer cells 48. The loss of *ARID1A* in pancreatic cancer cells correlates with poor differentiation and elevation of anti-apoptotic proteins, Bcl-2 49. In consistent, we found the decreased cell apoptosis in *ARID1A*-knockdowned Caco-2 cells. Furthermore, we also observed the increased cell proliferation in *ARID1A*-knockdowned cells. This data was in concordance with the previous evidence reporting the promotion of cell proliferation in *ARID1A* suppressive cells 50. A recent study also revealed that the promotion of CRC proliferation correlates with its aggressiveness in SOX12-overexpressed cells 51. These data underscore the role of *ARID1A* down-regulation in apoptosis resistance and enhancement of cell proliferation in CRC.

Another important characteristic of the cancer cells is its unlimited replication ability. Normally, the somatic cells has a limited lifespan - they can be replicated (but limitedly) and finally undergo cellular senescence, which is the process of permanent cell cycle arrest. Cellular senescence is a cell protective mechanism that can be detected by measuring the SA-β-gal activity 52, 53. Several lines of evidence have reported that suppression of cellular senescence is associated with the enhanced carcinogenesis 54, 55. Interestingly, several cancer cells can undergo senescence after exposure to chemotherapeutic agents 56-58. In CRC, recent studies have reported that the anti-tumor agents, e.g., baicalin, methotrexate, can induce cellular senescence 59, 60. Our data were in the same lines of those reported in previous studies. We observed the suppression of the cellular senescence activity of the cells by *ARID1A* knockdown, indicating the important role of *ARID1A* deficiency in the regulation of the replicative ability of the cancer cells.

Resistance to chemotherapy has been used to determine the ability of cancer cells to escape from growth suppressor signals in several cancer types 61-63. Our present study revealed the enhancement of the chemoresistance ability of the siARID1A-transfected cells. This data was consistent with those reported in several other studies demonstrating that the decreased expression of ARID1A is associated with chemoresistance in ovarian cancer and renal cell carcinoma 64, 65. In addition, the high level of ARID1A is found in paclitaxel-sensitive breast cancer cells, whereas its lower level is found in paclitaxel-resistant cells 66. These data highlight the important role of ARID1A deficiency in the chemoresistance of CRC.

We also found the greater invasive capability of the siARID1A-transfected cells. The spread of cancer cells from their origin to other organs or metastasis is the main cause of the cancer mortality. Invasion of the cancer cells is the main process underlying cancer metastasis, in which the cells are dissociated from their cell-cell adhesion and can then penetrate to other locales through the surrounding extracellular matrix (ECM). Several previous studies have shown that the loss or mutation of *ARID1A* tumor suppressor gene promote invasion of several cancers, including neuroblastoma 67, gastric cancer 68, hepatocellular carcinoma 69, and breast cancer 70. However, our present study showed the first evidence that could demonstrate the association between *ARID1A* deficiency and increased cell invasive capability of the CRC cells.

Two other phenotypes, which were examined in our study to evaluate the effects of *ARID1A* down-regulation on carcinogenesis and aggressiveness of CRC, were the PGCCs and multicellular spheroid formation. It has been previously reported that the PGCCs formation is associated with breast cancer cell invasion 71. The increase of the PGCCs number is also associated with the high grade of cancers 71-73. Furthermore, self-aggregation or multicellular spheroid formation has been considered as one of the parameters determining cancer aggressiveness 74. Interestingly, self-aggregation of the cancer cells promotes their survival and proliferation 75. However, the association between *ARID1A* deficiency and PGCCs as well as multicellular spheroid formation had not been previously studied. We report herein for the first time that both PGCCs and multicellular spheroid were increased by *ARID1A* knockdown in Caco-2 cells. These changes were in concordance with other carcinogenesis and aggressiveness features discussed above.

Induction of angiogenesis is another important hallmark of cancers and their aggressiveness. Due to the requirement for oxygen and nutrient supply for cellular activities, formation of the new blood vessels is needed. It has been reported that the growth of cancer cells without blood supply will be stopped when its size is over 1-2 mm^2^ in diameter 76. The angiogenesis is initially driven by secretion of angiogenic factors such as VEGF, angiopoietin and fibroblast growth factor from the cancer cells to recruit the endothelial cells to the cancer locale. It has been reported that expression levels of angiogenic factors can reflect the aggressiveness of cancers 77-79. In hepatocellular carcinoma, *ARID1A* deficiency promotes angiopoietin-2-induced angiogenesis that correlates with the cancer progression and aggressiveness 80. Interestingly, the most recent evidence has highlighted the direct effects of *ARID1A* down-regulation in human endothelial cells on angiogenesis via increased angiopoietin 2 secretion 81. Moreover, endometrioid carcinomas exhibit the loss of *ARID1A* with enhanced VEGF expression 82. Our data were consistent with those reported previously revealing that the *ARID1A*-knockdowned cells had increased VEGF secretion suggesting the potential of these cells with the *ARID1A* down-regulation to induce angiogenesis.

In summary, we have demonstrated that *ARID1A* knockdown suppresses cell death and cellular senescence in Caco-2 CRC cells. On the other hand, the *ARID1A* down-regulation enhances cell proliferation, chemoresistance, cell invasion, PGCCs formation, multicellular spheroid formation, and secretion of the angiogenic factor VEGF. These findings may, at least in part, explain the cellular mechanisms of *ARID1A* deficiency in carcinogenesis and aggressiveness features of CRC.

## References

[1] Hu G, Tu W, Yang L, Peng G, Yang L (2020). ARID1A deficiency and immune checkpoint blockade therapy: From mechanisms to clinical application. Cancer Lett.

[2] Wang L, Qu J, Zhou N, Hou H, Jiang M, Zhang X (2020). Effect and biomarker of immune checkpoint blockade therapy for ARID1A deficiency cancers. Biomed Pharmacother.

[3] Toumpeki C, Liberis A, Tsirkas I, Tsirka T, Kalagasidou S, Inagamova L (2019). The Role of ARID1A in Endometrial Cancer and the Molecular Pathways Associated With Pathogenesis and Cancer Progression. *In vivo*.

[4] Mittal P, Roberts CWM (2020). The SWI/SNF complex in cancer - biology, biomarkers and therapy. Nat Rev Clin Oncol.

[5] Clapier CR, Verma N, Parnell TJ, Cairns BR (2020). Cancer-Associated Gain-of-Function Mutations Activate a SWI/SNF-Family Regulatory Hub. Mol Cell.

[6] Ribeiro-Silva C, Vermeulen W, Lans H (2019). SWI/SNF: Complex complexes in genome stability and cancer. DNA Repair (Amst).

[7] Ge H, Xiao Y, Qin G, Gu Y, Cai X, Jiang W (2021). Mismatch repair deficiency is associated with specific morphologic features and frequent loss of ARID1A expression in ovarian clear cell carcinoma. Diagn Pathol.

[8] Xu G, Chhangawala S, Cocco E, Razavi P, Cai Y, Otto JE (2020). ARID1A determines luminal identity and therapeutic response in estrogen-receptor-positive breast cancer. Nat Genet.

[9] Nagarajan S, Rao SV, Sutton J, Cheeseman D, Dunn S, Papachristou EK (2020). ARID1A influences HDAC1/BRD4 activity, intrinsic proliferative capacity and breast cancer treatment response. Nat Genet.

[10] Yim SY, Kang SH, Shin JH, Jeong YS, Sohn BH, Um SH (2020). Low ARID1A Expression is Associated with Poor Prognosis in Hepatocellular Carcinoma. Cells.

[11] De P, Dey N (2019). Mutation-Driven Signals of ARID1A and PI3K Pathways in Ovarian Carcinomas: Alteration Is An Opportunity. Int J Mol Sci.

[12] Mathur R (2018). ARID1A loss in cancer: Towards a mechanistic understanding. Pharmacol Ther.

[13] Onder S, Fayda M, Karanlik H, Bayram A, Sen F, Cabioglu N (2017). Loss of ARID1A expression is associated with poor prognosis in invasive micropapillary carcinomas of the breast: A clinicopathologic and immunohistochemical study with long-term survival analysis. Breast J.

[14] Yang L, Wei S, Zhao R, Wu Y, Qiu H, Xiong H (2016). Loss of ARID1A expression predicts poor survival prognosis in gastric cancer: a systematic meta-analysis from 14 studies. Sci Rep.

[15] Sung H, Ferlay J, Siegel RL, Laversanne M, Soerjomataram I, Jemal A (2021). Global cancer statistics 2020: GLOBOCAN estimates of incidence and mortality worldwide for 36 cancers in 185 countries. CA Cancer J Clin.

[16] Kim YS, Jeong H, Choi JW, Oh HE, Lee JH (2017). Unique characteristics of ARID1A mutation and protein level in gastric and colorectal cancer: A meta-analysis. Saudi J Gastroenterol.

[17] Erfani M, Hosseini SV, Mokhtari M, Zamani M, Tahmasebi K, Alizadeh Naini M (2020). Altered ARID1A expression in colorectal cancer. BMC Cancer.

[18] Mathur R, Alver BH, San Roman AK, Wilson BG, Wang X, Agoston AT (2017). ARID1A loss impairs enhancer-mediated gene regulation and drives colon cancer in mice. Nat Genet.

[19] Wei XL, Wang DS, Xi SY, Wu WJ, Chen DL, Zeng ZL (2014). Clinicopathologic and prognostic relevance of ARID1A protein loss in colorectal cancer. World J Gastroenterol.

[20] Chou A, Toon CW, Clarkson A, Sioson L, Houang M, Watson N (2014). Loss of ARID1A expression in colorectal carcinoma is strongly associated with mismatch repair deficiency. Hum Pathol.

[21] Ye J, Zhou Y, Weiser MR, Gonen M, Zhang L, Samdani T (2014). Immunohistochemical detection of ARID1A in colorectal carcinoma: loss of staining is associated with sporadic microsatellite unstable tumors with medullary histology and high TNM stage. Hum Pathol.

[22] Baldi S, Khamgan H, Qian Y, Wu H, Zhang Z, Zhang M (2021). Downregulated ARID1A by miR-185 Is Associated With Poor Prognosis and Adverse Outcomes in Colon Adenocarcinoma. Front Oncol.

[23] Pongsakul N, Vinaiphat A, Chanchaem P, Fong-ngern K, Thongboonkerd V (2016). Lamin A/C in renal tubular cells is important for tissue repair, cell proliferation, and calcium oxalate crystal adhesion, and is associated with potential crystal receptors. FASEB J.

[24] Aluksanasuwan S, Sueksakit K, Fong-ngern K, Thongboonkerd V (2017). Role of HSP60 (HSPD1) in diabetes-induced renal tubular dysfunction: regulation of intracellular protein aggregation, ATP production, and oxidative stress. FASEB J.

[25] Fong-ngern K, Ausakunpipat N, Singhto N, Sueksakit K, Thongboonkerd V (2017). Prolonged K(+) deficiency increases intracellular ATP, cell cycle arrest and cell death in renal tubular cells. Metabolism.

[26] Kapincharanon C, Thongboonkerd V (2018). K(+) deficiency caused defects in renal tubular cell proliferation, oxidative stress response, tissue repair and tight junction integrity, but enhanced energy production, proteasome function and cellular K(+) uptake. Cell Adh Migr.

[27] Chen HY, Feng LL, Li M, Ju HQ, Ding Y, Lan M (2021). College of American Pathologists Tumor Regression Grading System for Long-term Outcome in Patients with Locally Advanced Rectal Cancer. Oncologist.

[28] Zhang S, Huang S, Zhang H, Li D, Li X, Cheng Y (2020). Histo- and clinico-pathological analysis of a large series of triple-negative breast cancer in a single center in China: Evidences on necessity of histological subtyping and grading. Chin J Cancer Res.

[29] Komatsubara T, Sakuma Y, Sata N, Fukushima N (2020). Histological evaluation of tumor differentiation score and prognosis of extrahepatic bile duct cancer: A proposal for a new histological grading system. Pathol Int.

[30] Bouris P, Manou D, Sopaki-Valalaki A, Kolokotroni A, Moustakas A, Kapoor A (2018). Serglycin promotes breast cancer cell aggressiveness: Induction of epithelial to mesenchymal transition, proteolytic activity and IL-8 signaling. Matrix Biol.

[31] Yonemori K, Seki N, Kurahara H, Osako Y, Idichi T, Arai T (2017). ZFP36L2 promotes cancer cell aggressiveness and is regulated by antitumor microRNA-375 in pancreatic ductal adenocarcinoma. Cancer Sci.

[32] Barresi V, Reggiani Bonetti L, Ieni A, Caruso RA, Tuccari G (2015). Histological grading in colorectal cancer: new insights and perspectives. Histol Histopathol.

[33] Fogh J, Fogh JM, Orfeo T (1977). One hundred and twenty-seven cultured human tumor cell lines producing tumors in nude mice. J Natl Cancer Inst.

[34] Cesna V, Sukovas A, Jasukaitiene A, Naginiene R, Barauskas G, Dambrauskas Z (2018). Narrow line between benefit and harm: Additivity of hyperthermia to cisplatin cytotoxicity in different gastrointestinal cancer cells. World J Gastroenterol.

[35] Kilic Suloglu A, Selmanoglu G, Akay MT (2015). Alterations in dysadherin expression and F-actin reorganization: a possible mechanism of hypericin-mediated photodynamic therapy in colon adenocarcinoma cells. Cytotechnology.

[36] Avivi-Green C, Polak-Charcon S, Madar Z, Schwartz B (2002). Different molecular events account for butyrate-induced apoptosis in two human colon cancer cell lines. J Nutr.

[37] Qiao L, Liu X, Tang Y, Zhao Z, Zhang J, Liu H (2018). Knockdown of long non-coding RNA prostate cancer-associated ncRNA transcript 1 inhibits multidrug resistance and c-Myc-dependent aggressiveness in colorectal cancer Caco-2 and HT-29 cells. Mol Cell Biochem.

[38] Dinicola S, Masiello MG, Proietti S, Coluccia P, Fabrizi G, Catizone A (2018). Nicotine increases colon cancer cell migration and invasion through epithelial to mesenchymal transition (EMT): COX-2 involvement. J Cell Physiol.

[39] Orlando A, Linsalata M, Russo F (2016). Antiproliferative effects on colon adenocarcinoma cells induced by co-administration of vitamin K1 and Lactobacillus rhamnosus GG. Int J Oncol.

[40] Gheytanchi E, Naseri M, Karimi-Busheri F, Atyabi F, Mirsharif ES, Bozorgmehr M (2021). Morphological and molecular characteristics of spheroid formation in HT-29 and Caco-2 colorectal cancer cell lines. Cancer Cell Int.

[41] Sanchez-Vega F, Gotea V, Chen YC, Elnitski L (2017). CpG island methylator phenotype in adenocarcinomas from the digestive tract: Methods, conclusions, and controversies. World J Gastrointest Oncol.

[42] Ahmed D, Eide PW, Eilertsen IA, Danielsen SA, Eknaes M, Hektoen M (2013). Epigenetic and genetic features of 24 colon cancer cell lines. Oncogenesis.

[43] Zhang X, Sun Q, Shan M, Niu M, Liu T, Xia B (2013). Promoter hypermethylation of ARID1A gene is responsible for its low mRNA expression in many invasive breast cancers. PLoS One.

[44] Roche ME, Lin Z, Whitaker-Menezes D, Zhan T, Szuhai K, Bovee J (2020). Translocase of the outer mitochondrial membrane complex subunit 20 (TOMM20) facilitates cancer aggressiveness and therapeutic resistance in chondrosarcoma. Biochim Biophys Acta Mol Basis Dis.

[45] Kaushik D, Ashcraft KA, Wang H, Shanmugasundaram K, Shah PK, Gonzalez G (2020). Nuclear NADPH oxidase-4 associated with disease progression in renal cell carcinoma. Transl Res.

[46] Shen B, Qian A, Lao W, Li W, Chen X, Zhang B (2019). Relationship between Helicobacter pylori and expression of programmed death-1 and its ligand in gastric intraepithelial neoplasia and early-stage gastric cancer. Cancer Manag Res.

[47] Zhang W, Liang X, Gong Y, Xiao C, Guo B, Yang T (2019). The Signal Transducer and Activator of Transcription 5B (STAT5B) Gene Promotes Proliferation and Drug Resistance of Human Mantle Cell Lymphoma Cells by Activating the Akt Signaling Pathway. Med Sci Monit.

[48] Wu S, Fatkhutdinov N, Fukumoto T, Bitler BG, Park PH, Kossenkov AV (2018). SWI/SNF catalytic subunits' switch drives resistance to EZH2 inhibitors in ARID1A-mutated cells. Nat Commun.

[49] Zhang L, Wang C, Yu S, Jia C, Yan J, Lu Z (2018). Loss of ARID1A Expression Correlates With Tumor Differentiation and Tumor Progression Stage in Pancreatic Ductal Adenocarcinoma. Technol Cancer Res Treat.

[50] Sun X, Chuang JC, Kanchwala M, Wu L, Celen C, Li L (2016). Suppression of the SWI/SNF Component Arid1a Promotes Mammalian Regeneration. Cell Stem Cell.

[51] Du F, Chen J, Liu H, Cai Y, Cao T, Han W (2019). SOX12 promotes colorectal cancer cell proliferation and metastasis by regulating asparagine synthesis. Cell Death Dis.

[52] Li K, Li Y, Yu Y, Ding J, Huang H, Chu C (2021). Bmi-1 alleviates adventitial fibroblast senescence by eliminating ROS in pulmonary hypertension. BMC Pulm Med.

[53] Zhuang Y, Li T, Xiao H, Wu J, Su S, Dong X (2021). LncRNA-H19 Drives Cardiomyocyte Senescence by Targeting miR-19a/socs1/p53 Axis. Front Pharmacol.

[54] Ramu D, Shan TW, Hirpara JL, Pervaiz S (2021). Cellular senescence: Silent operator and therapeutic target in cancer. IUBMB Life.

[55] Kolodkin-Gal D, Roitman L, Ovadya Y, Azazmeh N, Assouline B, Schlesinger Y (2021). Senolytic elimination of Cox2-expressing senescent cells inhibits the growth of premalignant pancreatic lesions. Gut.

[56] Jia M, Su B, Mo L, Qiu W, Ying J, Lin P (2021). Circadian clock protein CRY1 prevents paclitaxelinduced senescence of bladder cancer cells by promoting p53 degradation. Oncol Rep.

[57] Gu J, Wang J, Liu X, Sai K, Mai J, Xing F (2021). IL-6 derived from therapy-induced senescence facilitates the glycolytic phenotype in glioblastoma cells. Am J Cancer Res.

[58] Ye ZQ, Chen HB, Zhang TY, Chen Z, Tian L, Gu DN (2021). MicroRNA-7 modulates cellular senescence to relieve gemcitabine resistance by targeting PARP1/NF-kappaB signaling in pancreatic cancer cells. Oncol Lett.

[59] Wang Z, Ma L, Su M, Zhou Y, Mao K, Li C (2018). Baicalin induces cellular senescence in human colon cancer cells via upregulation of DEPP and the activation of Ras/Raf/MEK/ERK signaling. Cell Death Dis.

[60] Dabrowska M, Uram L, Zielinski Z, Rode W, Sikora E (2018). Oxidative stress and inhibition of nitric oxide generation underlie methotrexate-induced senescence in human colon cancer cells. Mech Ageing Dev.

[61] Lee M, Song Y, Choi I, Lee SY, Kim S, Kim SH (2021). Expression of HYOU1 via Reciprocal Crosstalk between NSCLC Cells and HUVECs Control Cancer Progression and Chemoresistance in Tumor Spheroids. Mol Cells.

[62] Kucuk B, Kibar B, Cacan E (2020). A broad analysis in clinical and *in vitro* models on regulator of G-protein signalling 10 regulation that is associated with ovarian cancer progression and chemoresistance. Cell Biochem Funct.

[63] Li Y, Liu H, Cui Y, Chen H, Cui X, Shao J (2020). miR-424-3p Contributes to the Malignant Progression and Chemoresistance of Gastric Cancer. Onco Targets Ther.

[64] Yokoyama Y, Matsushita Y, Shigeto T, Futagami M, Mizunuma H (2014). Decreased ARID1A expression is correlated with chemoresistance in epithelial ovarian cancer. J Gynecol Oncol.

[65] Somsuan K, Peerapen P, Boonmark W, Plumworasawat S, Samol R, Sakulsak N (2019). ARID1A knockdown triggers epithelial-mesenchymal transition and carcinogenesis features of renal cells: role in renal cell carcinoma. FASEB J.

[66] Lin YF, Tseng IJ, Kuo CJ, Lin HY, Chiu IJ, Chiu HW (2018). High-level expression of ARID1A predicts a favourable outcome in triple-negative breast cancer patients receiving paclitaxel-based chemotherapy. J Cell Mol Med.

[67] Li C, Xu ZL, Zhao Z, An Q, Wang L, Yu Y (2017). ARID1A gene knockdown promotes neuroblastoma migration and invasion. Neoplasma.

[68] Yan HB, Wang XF, Zhang Q, Tang ZQ, Jiang YH, Fan HZ (2014). Reduced expression of the chromatin remodeling gene ARID1A enhances gastric cancer cell migration and invasion via downregulation of E-cadherin transcription. Carcinogenesis.

[69] He F, Li J, Xu J, Zhang S, Xu Y, Zhao W (2015). Decreased expression of ARID1A associates with poor prognosis and promotes metastases of hepatocellular carcinoma. J Exp Clin Cancer Res.

[70] Takao C, Morikawa A, Ohkubo H, Kito Y, Saigo C, Sakuratani T (2017). Downregulation of ARID1A, a component of the SWI/SNF chromatin remodeling complex, in breast cancer. J Cancer.

[71] Fei F, Zhang D, Yang Z, Wang S, Wang X, Wu Z (2015). The number of polyploid giant cancer cells and epithelial-mesenchymal transition-related proteins are associated with invasion and metastasis in human breast cancer. J Exp Clin Cancer Res.

[72] White-Gilbertson S, Lu P, Jones CM, Chiodini S, Hurley D, Das A (2020). Tamoxifen is a candidate first-in-class inhibitor of acid ceramidase that reduces amitotic division in polyploid giant cancer cells-Unrecognized players in tumorigenesis. Cancer Med.

[73] Fei F, Zhang M, Li B, Zhao L, Wang H, Liu L (2019). Formation of Polyploid Giant Cancer Cells Involves in the Prognostic Value of Neoadjuvant Chemoradiation in Locally Advanced Rectal Cancer. J Oncol.

[74] Zhong Z, Pannu V, Rosenow M, Stark A, Spetzler D (2018). KIAA0100 Modulates Cancer Cell Aggression Behavior of MDA-MB-231 through Microtubule and Heat Shock Proteins. Cancers (Basel).

[75] Rayavarapu RR, Heiden B, Pagani N, Shaw MM, Shuff S, Zhang S (2015). The role of multicellular aggregation in the survival of ErbB2-positive breast cancer cells during extracellular matrix detachment. J Biol Chem.

[76] Nishida N, Yano H, Nishida T, Kamura T, Kojiro M (2006). Angiogenesis in cancer. Vasc Health Risk Manag.

[77] Saha A, Anirvan P (2020). Cancer progression in COVID-19: integrating the roles of renin angiotensin aldosterone system, angiopoietin-2, heat shock protein-27 and epithelial mesenchymal transition. Ecancermedicalscience.

[78] Li S, Wang L, Meng Y, Chang Y, Xu J, Zhang Q (2017). Increased levels of LAPTM4B, VEGF and survivin are correlated with tumor progression and poor prognosis in breast cancer patients. Oncotarget.

[79] Lin ZY, Chen G, Zhang YQ, He HC, Liang YX, Ye JH (2017). MicroRNA-30d promotes angiogenesis and tumor growth via MYPT1/c-JUN/VEGFA pathway and predicts aggressive outcome in prostate cancer. Mol Cancer.

[80] Hu C, Li W, Tian F, Jiang K, Liu X, Cen J (2018). Arid1a regulates response to anti-angiogenic therapy in advanced hepatocellular carcinoma. J Hepatol.

[81] Yoodee S, Peerapen P, Plumworasawat S, Thongboonkerd V (2021). ARID1A knockdown in human endothelial cells directly induces angiogenesis by regulating angiopoietin-2 secretion and endothelial cell activity. Int J Biol Macromol.

[82] Barreta A, Sarian LO, Ferracini AC, Costa LBE, Mazzola PG, de Angelo Andrade L (2019). Immunohistochemistry expression of targeted therapies biomarkers in ovarian clear cell and endometrioid carcinomas (type I) and endometriosis. Hum Pathol.

